# How does local government use the planning system to regulate hot food takeaway outlets? A census of current practice in England using document review

**DOI:** 10.1016/j.healthplace.2019.03.010

**Published:** 2019-05

**Authors:** Matthew Keeble, Thomas Burgoine, Martin White, Carolyn Summerbell, Steven Cummins, Jean Adams

**Affiliations:** a UKCRC Centre for Diet and Activity Research (CEDAR), MRC Epidemiology Unit, University of Cambridge School of Clinical Medicine, Box 285 Institute of Metabolic Science, Cambridge Biomedical Campus, Cambridge, CB2 0QQ, England, UK; b Fuse: the Centre for Translational Research in Public Health, Department of Sport and Exercise Sciences, Durham University, 32 Old Elvet, Durham, DH1 3HN, England, UK; c Department of Public Health, Environments & Society, Faculty of Public Health & Policy, London School of Hygiene and Tropical Medicine, 15-17 Tavistock Place, London, WC1H 9SH, England, UK

**Keywords:** Takeaway food outlet, Fast food, Food environment, Urban planning, Diet

## Abstract

Takeaway food outlets typically sell hot food, ordered and paid for at the till, for consumption off the premises due to limited seating provision. Growing numbers of these outlets has raised concerns about their impact on diet and weight gain. This has led to proposals to regulate their proliferation through urban planning. We conducted a census of local government areas in England with planning power (n = 325) to identify planning policies specifically addressing takeaway food outlets, with a ‘health’, and ‘non-health’ focus. We reviewed planning policies using content analysis, and developed a typology. One hundred and sixty-four (50.5%) local government areas had a policy specifically targeting takeaway food outlets; of these, 56 (34.1%) focused on health. Our typology revealed two main foci: ‘Place’ with five targeted locations and ‘Strategy’ with four categories of approach. The most common health-focused approach was describing exclusion zones around places for children and families (n = 33). Non-health focused approaches primarily involved minimising negative impacts associated with takeaway food outlets within a local government area boundary (n = 146). To our knowledge, this is the first census of planning policies explicitly focused on takeaway food outlets in England. Further work is required to determine why different approaches are adopted in different places and their acceptability and impact.

## Highlights

• We conducted a census of English local government areas (n = 325) to locate planning policies regulating takeaway food outlets.
• 164 (50.5%) local government areas had a policy specifically targeting takeaway food outlets, 56 (34.1%) focused on health.
• In a typology of approaches, we present two foci: ‘Place’ with five targeted locations and ‘Strategy’ with four categories.
• The most common health-focused approach was exclusion zones around places for children and families (n = 33).
• We found a range of current planning practice for regulating takeaway food outlets in England, more than previously estimated.

## 1 Introduction

The health implications associated with excess bodyweight, including type 2 diabetes, cardiovascular disease and several cancers, are well established (Reilly and Kelly, 2011; Swinburn et al., 2011). In England, around 60% of adults are overweight or obese, alongside 34% of children starting secondary school (National Health Service, 2017; Public Health England, 2018b). There are many determinants of obesity at individual, community, national and transnational levels (Swinburn et al., 1999, 2015), including physical access to neighbourhood food outlets.

Takeaway food outlets typically sell hot food, which is ordered and paid for at the till, for consumption off the premises due to limited seating provision (Burgoine et al., 2017). Foods served tend to be energy dense, high in total fats, saturated fats and salt (Jaworowska et al., 2014). The takeaway food industry in the United Kingdom has been valued at £8.9 billion, with strong predictions for further growth (Patterson et al., 2012). Access to, and use of, takeaway food outlets, may be an important determinant of subsequent unhealthy dietary behaviours and excess adiposity (Kirk et al., 2010). Across England, takeaway food outlet numbers have increased markedly in recent years. Between 2014 and 2017, the total number rose by 10% to nearly 58,000, while the proportion of all food outlets designated as takeaway food outlets also increased to around 27% (Food environment assessment tool (Feat), 2018). These trends are likely to have been mirrored across other countries (Lamb et al., 2017; Rodgers et al., 2018). Frequent use of takeaway food outlets has been associated with poorer diet and greater odds of obesity, with regular consumption of the typically energy-dense, and nutrient poor foods offered, linked over time to excess weight gain (Burgoine et al., 2014; Jaworowska et al., 2014; Pereira et al., 2005).

There have been attempts to make foods offered by takeaway food outlets healthier (Bagwell, 2014; Public Health England, 2018a). Although potentially effective, emerging evidence demonstrating the influence of the built environment on dietary behaviour, suggests that alternative, complementary regulatory approaches should also be explored. Urban planning refers to planned management of built environments, land use and development. Whilst we use the term ‘planning’ throughout, related terms used elsewhere include regional planning, land use and zoning. There has been international precedent for this. For example in the United States, where land use controls, or ‘zoning’ approaches have been used to promote active living (Chriqui et al., 2016). Elsewhere in the United States, the city of Los Angeles adopted a ‘fast food ban’ in 2008 (ordinance 180103), restricting new outlets from opening in designated exclusion zones (Los Angeles City Planning, 2008; Sturm and Hattori, 2015). More recently, the Irish Heart Foundation supported a proposed ‘no-fry zone’ around schools and other places often frequented by children (Irish Heart Foundation, 2016). Regulating the takeaway food sector to curb proliferation may serve as a low agency, population-level, public health intervention with potential impacts on diet and diet-related health inequalities (Adams et al., 2016b).

In England, the local planning system aims to ensure that communities benefit from appropriate development through determining acceptability of submitted planning applications (Department for Communities and Local Government, 2015). National guidance informs local level planning practice, and increasingly cites the potential role of the planning system to improve public health (Goodwin et al., 2014; Local Government Association, 2016; London Healthy Urban Development Unit, 2013; Ministry of Housing Communities and Local Government, 2017). For example, the National Planning Policy Framework states: “Planning policies and decisions should aim to achieve healthy, inclusive and safe places which: enable and support healthy lifestyles, especially where this would address identified local health and well-being needs – for example through the provision of … access to healthier food” (Ministry of Housing Communities and Local Government, 2018).

Local government areas (formally known as local authorities) are the lowest level of government in England, and all parts of the country are situated within one of these administrative boundaries. Each local government area has a number of administrative responsibilities. There are 353 in total, with 325 having responsibility for planning and, in some cases, public health. National guidance informs the content of Core Strategies (commonly known as Local Plans), Development Plan Documents and Supplementary Planning Documents (see Table 1 for definitions). These documents outline and justify planning criteria, which are used in the decision making process of determining acceptability of planning applications (Department for Communities and Local Government, 2012; Goodwin et al., 2014; Ministry of Housing Communities and Local Government, 2017).

**Table 1:** Description of English planning system documents.

Planning document	Description
Local Plans and Core Strategies	A document containing planning policies in line with the needs, concerns, priorities, vision, and strategic objectives of a local government area.
Development Plan Documents	Formal legal name for statutory planning documents including Local Plans, Core Strategies and Area Action Plans. Development Plan Documents may coexist with, or be a part of, a Core Strategy.
Supplementary Planning Document	A document providing additional detail, context and justification to a planning policy contained in a Development Plan Document. Supplementary Planning Documents must refer to an existing planning policy.

Commercial premises in England are designated a ‘Use Class’, which includes Class A5 retailers with the purpose of selling hot food for consumption off the premises (Town and Country Planning, 2005). Planning permission is required to open a new takeaway food outlet, and most often, planning permission is also required for a change of use class. It is at the point of planning permission application, that planning interventions to control takeaway food outlet proliferation apply.

There is anecdotal evidence of planning being increasingly used to regulate takeaway food outlets in England (Local Government Association, 2016; London Healthy Urban Development Unit, 2013). However, no systematic evidence has been published. Moreover, little is known regarding the variety of approaches taken, including whether and when diet, obesity or health are primary considerations. Such information could serve to inform future practice in England, as well as local, regional and national approaches elsewhere, while also helping to identify opportunities for evaluation. The purpose of this study was to conduct a census to identify the prevalence and nature of the use of planning to regulate takeaway food outlets across local government areas in England.

## 2 Methods

### 2.1 Planning policy document identification

Between November 2017 and March 2018, one researcher (MK) searched the websites of each local government area in England with planning power (n = 325, see Appendix 1), to identify planning policies within planning policy documents. Formally adopted documents were included, but draft documents containing details of future practice were not. Due to the study nature, searches focused exclusively on planning documents that would contain policies used to determine the acceptability of planning applications for new takeaway food outlets. Additional documents such as Joint Strategic Needs Assessments, which outline wider strategies to improve the health and wellbeing of local populations, would not be used in isolation to determine planning application acceptability, and were not included. Previous syntheses of ‘grey literature’ demonstrate systematic searches of websites as a means to identifying such documents (Adams et al., 2016a), and precedent has been set for the study of local government areas in England using this approach (Hillier-Brown et al., 2017).

To identify relevant documents we visited “Planning” and “Planning Policy” website sections of local government areas, and then located sub-sections referring to relevant documents described in Table 1. Searches initially focused on documents in these sections, and once identified they were either downloaded, or viewed online. Where a document was identified it was reviewed, and searched. Common terms associated with takeaway food outlets were used to search for planning policies referring to this type of outlet. These were: Use Class Order identifiers ‘A3’ (pre-2005) and ‘A5’ (2005 onwards), ‘hot food takeaway’, ‘fast food’, ‘health’, ‘diet’ and ‘obesity’. These search terms were used as they are common planning system terms in England, are used within planning policy guidance for local government areas, and reflect previous research in this area (Local Government Association, 2016; London Healthy Urban Development Unit, 2013; Turbutt et al., 2018). Where one search term failed to produce a result, a different term was used until all were exhausted, or a planning policy was identified. The number of times a search term was used, or the number of results a term generated, was not recorded as they were adopted for the purpose of planning policy identification rather than to determine terminology frequency. Where word based searches did not generate a result, document contents pages were reviewed to identify planning policies that explicitly named takeaway food, or hot food takeaway outlets in their titles. If this did not produce a result we visited “Retail” and “Shopping” sections of documents and reviewed each planning policy to identify references to takeaway food outlets.

At this stage, if a planning policy document contained a reference to takeaway food outlets in any way it was saved for later review. If a document cross-referenced additional planning policy documents in relation to takeaway food outlets, these documents were identified and reviewed. For example, to identify a cross-referenced Supplementary Planning Document, the ‘Supplementary Planning Documents’ sub-section of the respective planning policy website was visited and reviewed. When additional relevant documents were identified, the same searching and saving approach as described above was used. Planning and Planning Policy website sections, and sub-sections therein, were reviewed to the point of information and document saturation (Fusch and Ness, 2015).

If planning policies could not be identified during website searches and document reviews, we contacted relevant Planning or Planning Policy Departments by telephone. During telephone calls, we asked to speak with an individual responsible for planning policy management, development or implementation. Where telephone calls were unsuccessful, departments were emailed, unless specific contact details had previously been provided by telephone or identified in documents. In these cases individuals were contacted directly. After five business days a reminder email was sent. If after a further five business days a response was not received, a further email was sent to prompt a response.

To maximise consistency and replicability (Eileen and Kathy, 2011), regardless of contact method, local government areas were asked a standard question: ‘Does your local government area have an active planning policy designed specifically to address the numbers of Class A5 hot food takeaways for considerations of noise, litter, local character, public health or similar?’. This broad question was used because it would allow planning policies adopted for health or non-health reasons, as defined here, to be identified. If a planning policy was in place, local government areas were asked to provide a link to the full planning policy document.

### 2.2 Planning policy document review

Following identification of relevant documents, we completed a second, more detailed content review. We grouped planning policies into two groups: ‘specific’ planning policies that explicitly mentioned takeaway food outlets either in their title, supporting text or planning policy criteria; and ‘non-specific’ planning policies that had no explicit focus on takeaway food outlets but could apply to takeaway food outlet planning applications. Non-specific planning policies typically addressed all retail outlets, and were not considered for further review.

Planning policies can contain multiple planning criteria, which describe standards that submitted applications are assessed against. Planning criteria were used to determine local government area focus, splitting those with and without an explicit focus on diet, obesity or diet-related disease, as ‘health’ or ‘non-health’, respectively. If at least one planning criteria within a planning policy was ‘health’ focused, the overall policy, and therefore local government area, was considered to have a health-focused takeaway food outlet planning policy. Regardless of health or non-health focus, all specific planning policies and planning criteria were included in the next stage of analysis.

### 2.3 Thematic content analysis

We read and re-read planning policies and associated planning criteria, as many times as necessary, and tracked key characteristics as they emerged (Braun and Clarke, 2006, 2013). Ongoing, iterative, thematic content analysis was used to identify key themes, which were agreed by all members of the study team (Braun and Clarke, 2006; Colorafi and Evans, 2016). We chose this approach due to the innovative nature of this work. Adopting an *a priori* approach would not have been appropriate due to a lack of existing evidence to guide analysis (Stuckey, 2015). We aimed to minimise the number of themes, whilst maximising differences between them; this allowed us to organise similar strategies within themes. Analysis focused on a literal interpretation of the published word, with no further interpretation (Elo et al., 2014; Vaismoradi et al., 2016).

## 3 Results

### 3.1 Planning policies

**Fig. 1:**
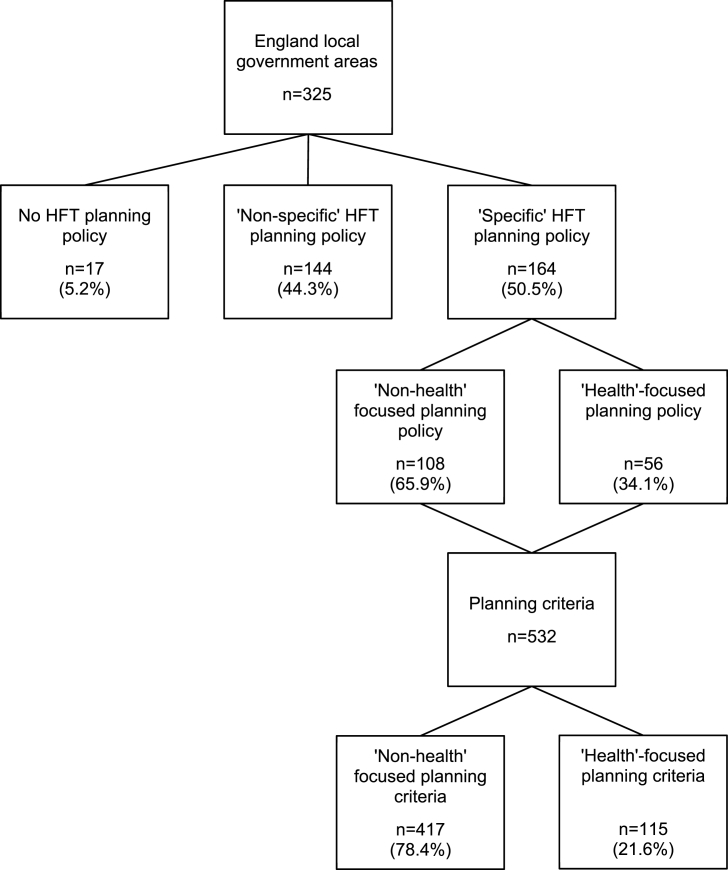
Planning policies identified at local government area level, specific to hot food takeaway (HFT) outlets, with a health or non-health focus. Data are expressed as the number (count) of observations, with percentages from the preceding number in the hierarchy.

From 325 local government areas with planning powers (Fig. 1), 17 (5.2%) did not have a planning policy related to takeaway food outlets and 144 (44.3%) had non-specific policies. Of 164 local government areas with specific planning policies, 56 (34.1%) were health-focused and 108 (65.9%) were not. Across these 164 local government areas, planning policy documents contained 532 individual planning criteria, of which, 115 (21.6%) were health-focused, 417 (78.4%) were not.

**Fig. 2:**
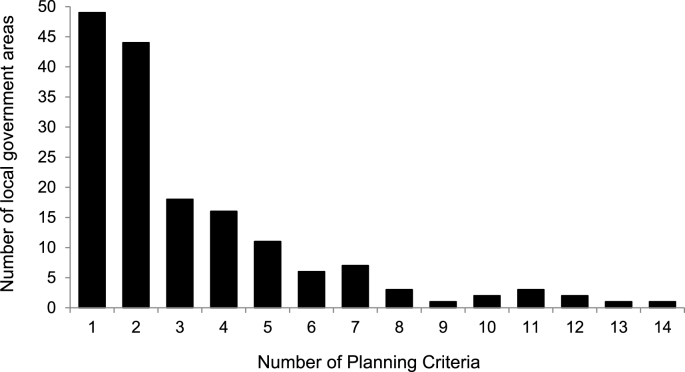
Distribution of number of planning policy criteria across local government areas (n = 164) with ‘specific’ takeaway food outlet planning policy.

Planning policy documents frequently contained multiple planning criteria. Fig. 2 shows the distribution of the number of planning criteria per local government area (n = 164), in specific takeaway food outlet planning policies. The median number of planning criteria was 2 (IQR 1–4), with 10% of local government areas containing 7 or more.

### 3.2 Nature of specific planning policies

Thematic analysis of specific planning policy criteria identified two important ‘axes’ of action: ‘Place’, with five locations of focus, and ‘Strategy’ with four approach categories, leading to 20 possible domains of action. Table 2 describes the locations of focus on the Place axis, and Table 3 describes categories of approach on the Strategy axis. We combined these axes to form a two-dimensional typology.

**Table 2:** Description of ‘Places’ on the typology's vertical axis.

Place	Description
All Areas Within a Local Government Area Boundary	Planning criteria applied to all proposals within a local government area, regardless of specific location of proposed takeaway food outlet site
Immediate Vicinity of Existing Hot Food Takeaway Outlet Site	The area immediately surrounding existing takeaway food outlet sites
Places for Children & Families	Locations commonly used or attended by, young children accompanied by family members, and/or older children independently
Retail Areas	Designated retail zones. Sometimes referred to as ‘high-streets’
Residential Areas	Designated zones, mostly comprising residential housing

**Table 3:** Description of ‘Strategies’ on the typology's horizontal axis.

Strategy	Description
Exclusion Zones	Opening of new takeaway food outlets will be restricted within these zones
Limit Density	Opening of new takeaway food outlets will be restricted, where numbers exceed stated acceptable thresholds
Minimise Impact and Protect Vicinity	Potentially negative consequences associated with takeaway food outlet operation are to be minimised
Other Strategies	Other approaches not common enough to receive individual classification

**Fig. 3:**
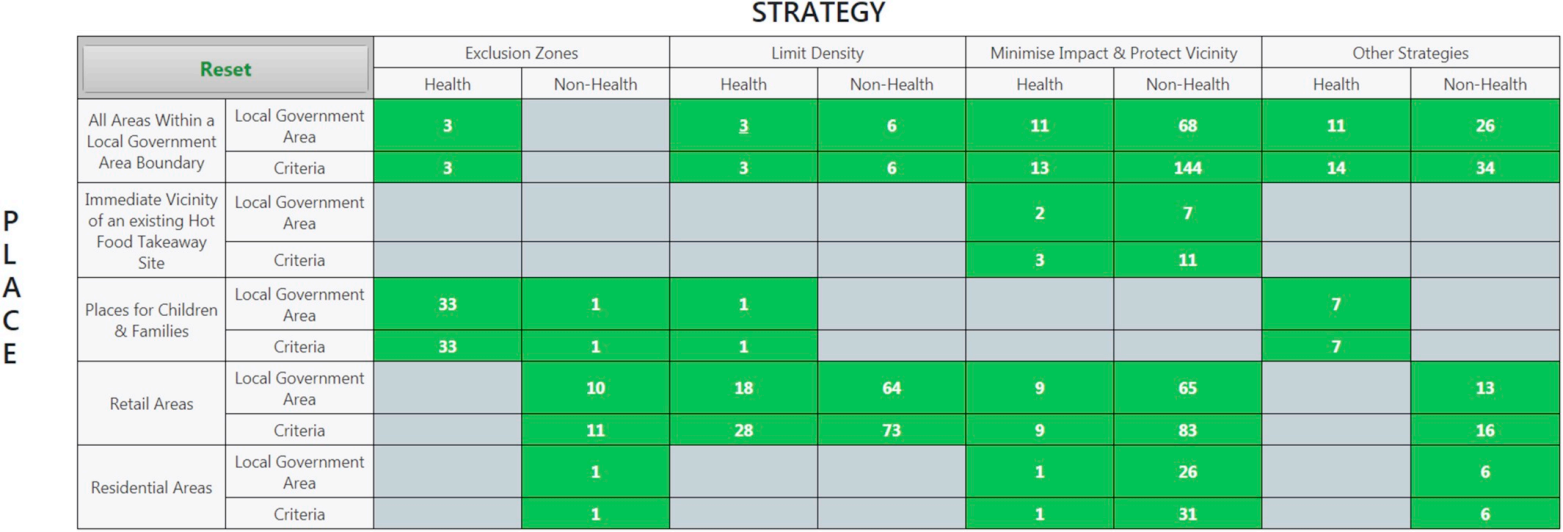
Typology of English local government area takeaway food outlet planning regulation. Level 1 of the typology (as shown) displays counts of planning policy criteria and contributing local government areas stratified by ‘health’ and ‘non-health’ focus, across two axes of action: Place and Strategy. Grey domains represent no action. Levels 2 and 3 are accessible via an interactive version of this typology, available here: https://hft-tool.mrc-epid.cam.ac.uk/.

We present a static version of level 1 of our typology in Fig. 3, showing the number of adopted planning policy criteria within each domain, alongside the number of local government areas across which these planning criteria are distributed, stratified by health or non-health focus. Shaded grey areas are domains with no current action.

Levels 2 and 3 of our typology are accessed online (see hyperlink in Fig. 3 caption). Level 2 provides paraphrased descriptions of adopted planning criteria within each domain. Level 3 shows full, verbatim, planning policy criteria within each domain, and also provides links to local government area websites to view full planning policy documents, allowing planning criteria to be viewed alongside supporting justification and in the context of the original planning policy document.

### 3.3 Health-focused planning criteria

The most commonly represented health-focused domain of action was exclusion zones near places for children and families (n = 33 (28.7%)) e.g. schools, parks and leisure facilities including sport centres and youth clubs. The details of exclusion zone policies differed by local government area. Distance based exclusion zones ranged from 200 to 800 m, and walking time based exclusion zones ranged from 5 to 10 min. Other strategies addressing places for families and children included restriction of takeaway food outlet opening hours during school lunch times, and immediately after school.

The second most commonly represented health-focused planning policy criteria domain were attempts to limit density of takeaway food outlets in retail areas (n = 29 (25.2%)). This included limiting the maximum number of consecutive takeaway food outlets, or capping the proportion of all retail space occupied by this use, with thresholds of 5–20%. Criteria designed to minimise impact, and a range of other strategies, implemented across entire local government area boundaries, were the third most commonly represented domains (13 criteria (11.3%) each). Other domains where health-focused action was identified included; implementation of community infrastructure levies with funds allocated to obesity prevention initiatives; mandatory sign-up to a healthy catering commitment scheme; and requirements for submission of health impact assessments alongside planning applications.

Three local government areas described the potential for exclusion zones across their full geographical area depending on childhood obesity rates for those in the first (aged 4–5) or last (aged 10–11) year of primary school. New takeaway food outlets would not be allowed if rates exceeded a given threshold (e.g. 15% of children in last year of primary school with excess weight) or the national average.

No local government areas described exclusion zones within the immediate vicinity of existing hot food takeaway outlet sites, or within retail or residential areas, and none had a planning policy to limit takeaway food outlet density within the immediate vicinity of existing hot food takeaway outlet sites, or within residential areas.

### 3.4 Non-health focused planning criteria

Amongst those with a non-health focus, the most commonly represented planning policy criteria described minimising the impact, and protecting the immediate vicinity, of an area from new takeaway food outlets. Planning criteria frequently aimed to minimise litter, smells, noise, traffic and anti-social behaviour, and often referred particularly to waste disposal, extraction equipment, security shutters and other design features, which were regulated with the aim of protecting the character or aesthetic appeal of an area. These planning criteria were most commonly applied across all parts of a local government area (n = 146 (35.0%)), or within retail areas (n = 83 (19.9%)).

The next most common domain represented at the planning criteria level, was to limit takeaway food outlet density in retail areas (n = 73 (17.5%)). Strategies were similar to health-focused approaches, and included imposing a maximum number of consecutive outlets (1–4), and restricting outlet number where there was less than a 10 m radius between one another. One local government area described an exclusion zone around places for children and families for non-health reasons, but no local government areas described exclusion zones or aimed to limit takeaway food outlet density in the immediate vicinity of existing takeaway food outlet sites, for this reason.

## 4 Discussion

### 4.1 Summary of findings

We found that 164 (50.5%) of 325 local government areas in England, with local planning power, had planning policies specific to takeaway food outlets. Of these, 56 (34.1%) had an explicit health focus. Across 164 local government areas with a specific planning policy, we found 532 individual planning criteria, of which 115 (21.6%) were health-focused. Content analysis revealed common places and strategies within adopted planning policy criteria; these were used to build a typology with two axes. Among planning criteria related to health, the most common approach described exclusion zones around places used by children and families, followed by approaches limiting density in retail areas. The most common non-health-focused approaches were those aiming to minimise the impact of outlets on local places, implemented across entire local government areas.

### 4.2 Interpretation of findings

Our results demonstrate that use of the planning system for takeaway food outlet regulation is more common than indicated in previous academic work. Previous work suggested that around 20 local government areas in England had planning policies for takeaway food outlet regulation, through adoption of Supplementary Planning Documents (Lake, 2018). We found 51 areas with Supplementary Planning Documents or other planning practice guidelines that made specific reference to takeaway food outlets. More broadly, we found that 164 areas had a planning policy specifically aimed at takeaway food outlet regulation. This focus on takeaway food outlets is in line with guidance and recommendations in the latest National Planning Policy Framework (Ministry of Housing Communities and Local Government, 2018).

There are several possible explanations for previous underestimates of policy action in this arena. Firstly, not all planning policies we identified were in Supplementary Planning Documents. This means that previous focus on these types of documents, for example in planning practice reviews (Health Planning and Sustainability, 2018), may have resulted in underestimations. Secondly, as ‘hot food takeaways’ did not always feature in planning policy titles, our detailed search of full documents may have helped find a wider range of approaches. Thirdly, we found substantial numbers of planning policies for takeaway food outlet regulation that did not include a health focus but may nevertheless have health impacts. It is possible that previous estimates had adopted a more health-specific approach. Lastly, local government use of planning to regulate takeaway food outlets has to date, largely been documented in the form of isolated case studies. For example, Waltham Forest and Barking and Dagenham local government areas are commonly cited (London Borough of Barking and Dagenham, 2010; London Borough of Waltham Forest, 2009). Elsewhere, Gateshead Metropolitan Borough Council gained attention for winning a Local Government Association award for Public Health (Department for Communities and Local Government, 2012; London Healthy Urban Development Unit, 2013; Ministry of Housing Communities and Local Government, 2017). It is possible that repeated citing of a small number of case studies may have further reinforced the impression that planning policy adoption, for the purpose of takeaway food outlet regulation, is uncommon. We have demonstrated this is not the case.

Our work demonstrates that local government areas adopt a variety of approaches within planning policies. There can be important lessons learned, and suggestions taken, from successful case studies to help direct planning practice. However, relying on a small range of case studies does not allow the full extent of possible approaches to be appreciated. Case studies also imply a ‘one-size-fits-all’ approach to regulation, when a wider range of practice is both possible and enacted. For example, as previously described, local government areas appear to converge on 400 m and 800 m exclusion zones around schools (Public Health England, 2014), however, these distances may not be appropriate in all local contexts. Meanwhile, our 20-domain typology emphasises a range of alternative strategies which may be more appropriate. We also found no adopted approaches in five typology domains, indicating potential for further policy innovation. While some strategies were common, some local government areas demonstrated innovation by adapting and evolving existing approaches. For example, three local government areas in the North East of England (Gateshead Metropolitan Borough Council, 2015; North Tyneside Council, 2017; South Tyneside Council, 2017) described exclusion zones based on contemporaneous childhood obesity rates from the National Child Measurement Programme, as opposed to rates at the time of intervention adoption. These policies represent a dynamic approach towards takeaway food outlet regulation. They also show local government areas taking local and national contexts into consideration to adopt approaches that best address their needs. Within a Government commissioned review, it was suggested that the English planning system had become too reactive (Farrell, 2014). Policies of this nature suggest however that it can be used proactively.

We found that almost 80% of takeaway food outlet planning criteria were not health-focused. Future research should explore the extent to which this imbalance is purely a historical artefact (i.e. arising from a focus on diet and obesity that has emerged only recently, in line with guidance), or for example, whether non-health rationale are more acceptable to relevant stakeholders and less likely to be challenged. The imbalance between health and non-health focus of planning criteria could also result from an existing belief that planning should not be adopted for the purposes of improving public health. This is reflected in previous work suggesting that employees within Planning Departments do not always see health promotion as part of their role, or of the planning system more broadly (Lake et al., 2017). This is despite responsibility for public health residing at local government level in England, and guidance stating that the planning system should be used to promote public health (Heath, 2014; Ministry of Housing Communities and Local Government, 2018). This is not to say that non-health focused planning criteria will not yield health impacts. For example, regulating the proliferation of takeaway food outlets to protect the character of local areas may well have similar impacts as regulating proliferation to reduce the burden of obesity.

Hot food takeaway outlets tend to cluster together, and in close proximity to, for example, schools and town centres (Ellaway et al., 2012). This may explain why policies addressing their presence in these areas emerged most strongly during the development of our typology. In contrast, there were no examples of policies focusing on density solely in residential areas. To some extent, this makes sense, reflecting the low number of takeaway food outlets in these areas (Ellaway et al., 2012). However, public health may be well served by addressing takeaway food outlets, for example, in residential areas, where greater access has been associated with poorer diet and increased obesity risk (Cobb et al., 2015; Cummins and Macintyre, 2006; Sisnowski et al., 2017).

### 4.3 Methodological considerations

We adopted a systematic approach to identify documents containing applicable planning policies, minimising the risk of selection bias. Local government areas must make all planning policy documents available online, meaning our primary reliance on websites is likely to be highly sensitive. Where a planning policy could not be identified, local government areas were contacted by telephone initially and then email. This allowed us to locate relevant planning policy documents that may have been inadvertently missed during website reviews.

By conducting a census of all local government areas our results are generalizable to the whole of England. The planning system and the process of planning policy adoption vary internationally, and our results are unlikely to be representative beyond England. However, the range of approaches identified may still be relevant to other locations considering the use of planning for takeaway food outlet regulation.

The major limitation of our study is its cross-sectional design within the context of an ever-changing planning system. During data collection we noted that around 70 local government areas had published draft planning policy documents that included references specifically to takeaway food outlets. These emerging policies may now have been adopted, but are not included in our census. Similarly, previous policies that were adopted but withdrawn prior to our census will not have been included.

We used a conventional content analysis approach (Colorafi and Evans, 2016), where planning criteria were interpreted literally without consideration of underlying policy intent or rationale. It is possible, for example, that some local government areas have a primary intent to protect health, but describe non-health focused planning policies as this is more politically acceptable.

### 4.4 Implications for policy, practice and future research

Our research highlights that local government use many and varied approaches within the planning system to regulate takeaway food outlets, with both health and non-health foci. Future research could explore why multiple, often similar, approaches are adopted. The acceptability of such approaches to a range of local stakeholders may also be an important barrier to adoption of new planning policies. Intervention acceptability should be a topic of further research. Furthermore, future research should seek to understand who the key actors and decision-makers are in planning policy development. This may provide those in local government who are looking to develop planning policies for takeaway food outlet regulation with guidance on how best to navigate this process. Moreover, whilst we have identified that planning policies have been adopted, their implementation (a precursor for long-term effectiveness) is unknown, and this should be explored in future research. Related to this, little is known about the impact of planning policy adoption on health, and other outcomes. Further work is urgently required to determine which approaches are likely to be most effective. Analysis of the 2008 Los Angeles ‘fast food ban’ suggested that regulation in this context may not have achieved the desired outcomes (Sturm and Cohen, 2009). It is important to acknowledge that whilst planning can control the development of food retail in an area to an extent, it is unable to control the foods served (Neckerman, 2014), and that complementary interventions designed to improve the healthiness of foods served within stores are likely to be necessary (Bagwell, 2014).

## 5 Conclusion

In this census of current planning policy in England, we found that around half of local government areas have a planning policy specific to takeaway food outlet regulation, with around a third of these including a health focus. We categorised approaches into a two-dimensional, 20 domain, typology. Existing approaches fall within 15 domains, indicating a wide range of current approaches, and further potential for novel policy development. Further work should explore determinants and impacts of different policies. Local government areas can use our typology to explore the range of policies and associated criteria already in place when seeking to implement their own planning policy for takeaway food outlet regulation.

## Authors' contributions

The study was devised by JA and TB. MK completed data collection and led data analysis, in consultation with JA, TB, MW, CS and SC. JA, TB and MK drafted the manuscript together. All authors read and approved the final manuscript.

## Figures and Tables

**Figure:**
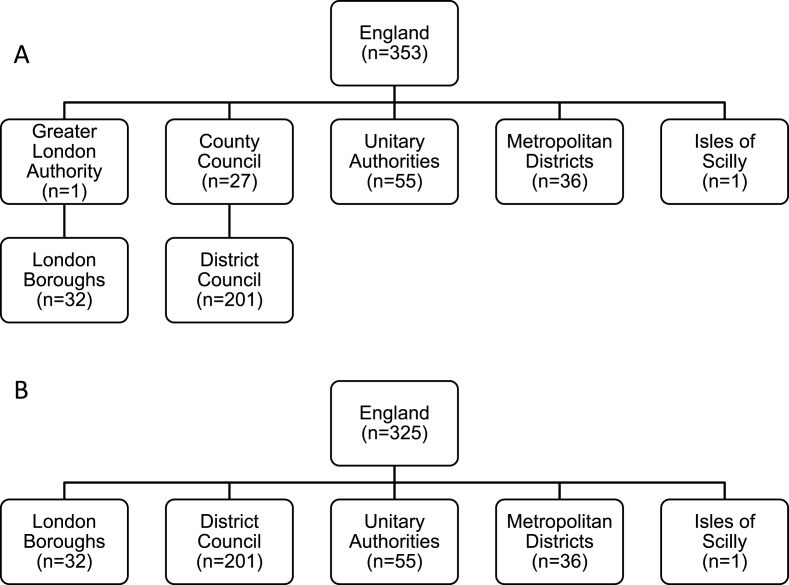


## Appendix 1

English local government structure. A) Number of each type of local government area in England. B) Number of each type of lower-tier local government area, with planning power in England, and therefore included in our census.
